# Prediction of delay discounting in intertemporal decisions by future thinking: Accounting for fluency, contents, and functions of future thoughts

**DOI:** 10.1002/brb3.2764

**Published:** 2022-09-19

**Authors:** Fatemeh Eivazi, Javad Hatami, Alireza Moradi, Mohammad‐Reza Nazem‐Zadeh

**Affiliations:** ^1^ Shahid Beheshti University, Institute for Cognitive and Brain Sciences Tehran Iran; ^2^ Department of Psychology, Faculty of Psychology and Education University of Tehran Tehran Iran; ^3^ Department of Clinical Psychology, Faculty of Psychology and Education Kharazmi University Tehran Iran; ^4^ Tehran University of Medical Sciences, Research Center for Molecular and Cellular Imaging, Advanced Medical Technologies and Instruments Institute (AMTII) Tehran Iran; ^5^ Physics and Biomedical Engineering Department Tehran University of Medical Sciences Tehran Iran

**Keywords:** delay discounting, future cognition, future thinking, intertemporal decision‐making

## Abstract

**Purpose:**

To study the variations of delay discounting rates as a function of fluency, contents, and functions of future thoughts in healthy subjects.

**Background:**

Delay discounting (DD) is a concept that can measure a frequent tendency toward smaller, yet immediate rewards, while a delayed reward is greater in value. DD describes people's choices in intertemporal decisions and is associated with self‐control. Future thinking (FT) and having a vivid imagination of the future can reduce individuals’ DD rates. However, constructing a specific episodic future representation was merely studied in relation to DD. Although fluency and contents of future thoughts have been reported related to various disorders and behaviors, their association with DD has not been previously addressed.

**Methods:**

The present study applies a verbal fluency task named the personal future task (PFT), the functions of future thinking scale (FoFTS), and the 27‐item delay discounting questionnaire (DDQ) in order to assess fluency, contents, and functions of future thoughts, and delay discounting in healthy subjects (*N* = 114, Female = 64%, Male = 36%, Mage = 34.22, SDage = 7.15).

**Results:**

Findings indicate that fluency of future thoughts is associated with DD. Among the contents of FT categories, financial contents (future thoughts about money and real estate matters), and regarding functions of FT, engaging in FT for planning are related to DD. Due to the final model, the above‐mentioned correlated variables can be considered as significant predictors of intertemporal choices when controlling for education and gender (*R* ^2^ = 0.4, Adjusted *R* ^2^ = 0.33, *F* = 5.186, *p*‐value = 0.001).

**Conclusion:**

The frequency of future thoughts one can generate, specifically future thoughts about financial contents, is associated with less short‐sighted intertemporal decisions. The former relationship is enhanced for longer delays (e.g., 5–10 years). Besides, individuals who frequently engage in FT for planning (planning out sequences of actions) discount future rewards to a lesser extent.

## Graphical Abstract

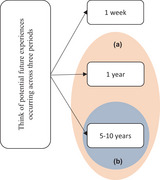

## 1 INTRODUCTION

Attaining long‐term goals or mastering major challenges often demands a high amount of patience. It implies that retreating from a desire for immediate pleasure is essential to get a greater reward in the future (Göllner, et al., 2018). Numerous daily decisions require individuals to alternate between the two options of a larger delayed reward over a smaller immediate one.

Although a larger delayed reward is of higher value, there is a tendency toward discounting its value in favor of the smaller immediate reward. This process is known as delay discounting (DD) or temporal discounting (Peters and Büchel, 2011), which describes how individuals make intertemporal decisions, referring to a decline in subjective values of an outcome when it is delayed (Mazur, 1987). It is typically measured using specific tasks providing a series of choices to determine the participant's preference toward a smaller immediate reward or a larger, but delayed alternative (Madden & Johnson, 2010).

This concept is reliably related to subjective self‐control or impulsiveness and plays an important role in daily life choices in concordance with healthy and addictive behaviors (Green & Myerson, 2004). On the other hand, choosing a larger delayed reward can be associated with many positive outcomes in personal life such as improvements in academic performance, physical health, and vigorous relationships (Hirsh et al., 2008). Therefore, exploring factors to reduce discounting rates can be beneficial for studying various aspects of human life.

Episodic future thinking (EFT) has been known as an effective means to alter individuals’ temporal perspective toward larger delayed rewards and less discounting deeds (Dassen, et al., 2016a; Nan & Qin, 2019; O'Donnell, et al., 2019, 2017; O'Neill et al., 2016; Stein et al., 2017, 2016, 2021; Rosch et al., 2022). Episodic future thinking is a cognitive ability to vividly simulate life events that will probably occur in the future (Atance & O'Neill, ). Engaging in EFT while making intertemporal decisions reduces the DD (Peters & Büchel, 2010) so that the persons choose in favor of their future benefits rather than present rewards (Chapman, 2005; Schacter et al., 2017). Future simulation provides a motivational break that confronts intrinsic desires toward short‐sighted decisions (Boyer, 2008). The effect of EFT on DD has been studied in eating behaviors, reporting a substantial reduction in calorie intake (Dassen, et al., 2016b; O'Neill et al., 2016; Stein et al., 2017). Other studies have shown a similar effect in attenuating smoking and alcoholic habits (Nan and Qin, 2019; Mellis et al., 2019; Snider et al., Bickel, 2016; Stein et al., 2016). Despite the wide stream of research mentioned earlier in which EFT influences DD only when the future cue is presented to the participant when making the intertemporal decision, Bromberg et al. (2015) showed individual differences in EFT are related to individual differences in adolescents (range 12–16 years). They claimed EFT capabilities of adolescents can contribute to their discounting behaviors (Bromberg et al., 2015). Further investigations may be beneficial in this regard.

Besides, some studies unraveled that the majority of episodic future thoughts come to our minds directly and rapidly without much effort and information manipulation (Cole & Kvavilashvili, 2021; Jeunehomme & D'Argembeau, 2016). Constructing a specific effortful episodic future representation is merely employed when constrained to the imagination of a novel experience (Jeunehomme & D'Argembeau, 2016). However, only the specificity of future thoughts has been investigated in the assessment of the relationship between EFT and DD (Bromberg et al., 2015). Therefore, in order to broaden our understanding of individual differences in FT contributing to DD, also to generalize and internalize the influence of FT on DD in daily life, it might be valuable to consider the direct production of future thoughts, which can be investigated through a fluency task and measuring accessibility of future events (MacLeod, et al., 1997). This is an abbreviated FT task applicable for clinic or research.

Moreover, D'Argembeau et al. (2011) showed that individuals may vary based on the thematic contents of their future‐oriented thoughts including work, leisure activities, relationships, and errands. Other studies demonstrated that different contents of future thought affect maladaptive behaviors and disorders (Bouwman, 2016; Godley et al., 2001).

According to the literature, despite a fair amount of research conducted in this field, there still seems to lack of focus on the variability that fluency and contents of future thoughts would impose on DD. Therefore, the aim of the present study was to investigate whether the DD rates vary as a function of fluency and thematic contents of future thoughts. The predictive utility of the afore‐mentioned variables over the DD rates was also of great interest. It was hypothesized that DD would be predicted by FT. Regarding the contents of future thoughts, it was also hypothesized that the achievements category would be negatively associated with DD due to the achievements’ goal‐oriented nature. O'Donnell et al. (2017) showed that goal‐oriented EFT cues caused more reduction in the DD rates than general ones. Besides, achievements in education require patience toward delayed rewards (Jaroni, et al., 2004). In order to take a broader perspective toward FT, the individual characteristics of engagement in FT were also measured (FoFTS; Hallford & D'Argembeau, 2020).

## 2 METHOD

### 2.1 Participants

In total, 114 adults (female = 64%, male = 36%) aged 22−50 years (M = 34.22, SD = 7.15, Median = 34) were recruited. The sample size was based on the minimum required for a multiple regression study, given the desired probability level (0.05), employed predictors in the final model, a medium effect size (0.15), and a desired statistical power level (0.9) suggested by Cohen (1988). Participants were notified of this experiment via social media and the laboratory recruitment system. Interested subjects were screened for any mental health issues.

The analyses indicated that no participant showed a consistency rate under 0.75 for an assigned *k*‐value, therefore, all of them were retained in the study (Kaplan et al., 2016).

### 2.2 Materials

#### 2.2.1 Delay discounting task

The 27‐item delay discounting questionnaire (DDQ) by Kirbyet al. (1999) was applied to measure delay discounting behaviors. In the monetary‐choice questionnaire for each item, the participant chooses between a smaller, yet immediate monetary reward; and a larger, but delayed one. Delays ranged between 7 and 186 days.

The participant's discounting behavior can be described by the following hyperbolic function:

(1) $$V=A/\left(1+kD\right)$$

Where *V* is the present value of the delayed reward *A* at the delay time *D*, and *k* is the discounting rate, typically ranges between 0.0 and 0.5, where the smaller values indicate a lack of discounting in favor of delayed rewards; while the higher values suggest a strong discounting and a preference for immediate rewards, which may recommend a high degree of impulsivity (Mazur, 1987).

#### 2.2.2 Personal futures task (PFT)

Future thinking was assessed by the personal future task (PFT) (MacLeod et al., 1997). The participants were required to think of potential future experiences occurring across three periods: the next week (including today), the next year, and the next 5–10 years. The periods were presented one at a time and in the order given above. The participants had 1 minute at each period to generate as many answers as possible, which were recorded. It had been explained to them that it would not matter if the events they were thinking of were unimportant, but they should be likely to happen. They were instructed to keep thinking until the end of the experiment time.

Before the administration of the FT task, the participants were given a standard verbal fluency task (to control for general cognitive fluency), in which they were instructed to generate as many words as they could in 1 minute, beginning with a certain letter of the alphabet (i.e., S and A), not including any proper nouns. They were also requested to name aloud as many animals as they could in 1 minute .

The contents of future thoughts were categorized according to the coding system introduced by Godley et al. (2001), with minor modifications (depicted in Table 1).

**TABLE 1:** Coding system for contents of future thoughts

Social	Social or interpersonal items that involve at least one other person.
Achievement	Academic and job‐related achievements. Other kinds of exams or certifications are also included.
Intrapersonal	Items that concern personal matters and not others. Like depression, personal development, etc.
Leisure	Events done for pleasure or leisure.
Health	Items relating to the subjects’ health and fitness.
Errands	Items relating to daily activities.
Financial	Money and real estate matters.

**FIGURE 1:**
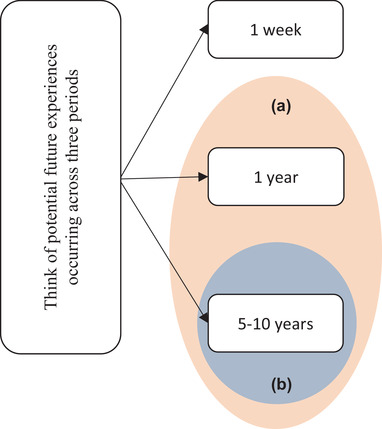
The PFT Delays were categorized as depicted for further analysis: (A) Distant delays. (B) 5–10 years

Since distant delays may add supplementary findings to the study, the sum of the total number of personal future thoughts and future thoughts in each category within three delays (1 week, 1 year, 5–10 years), two distant delays (1 year, 5–10 years), and also the longest delay (5‐10 years) were assessed (Figure 1).

#### 2.2.3 The Functions of Future Thinking Scale (FoFTS)

The 30‐item FoFTS scale is adopted (Hallford & D'Argembeau, 2020) to evaluate the frequency of self‐reported functions of FT consisting of the 10 functions mentioned below:

Boredom reduction, death preparation (anticipating and adjusting to the idea of death), identity contrasting (thinking about the type of person one might want to be), negative emotion regulation (downregulating negative emotion concerning upcoming experiences), social bonding (increasing closeness with others), goal setting, planning (planning out sequences of actions), problem‐solving (solving future problems), decision‐making (thinking of outcomes of made decisions), and positive emotion regulation (upregulating positive emotions). This scale measures the main functions that induce FT in individuals.

### 2.3 Procedure

Each interview session started with a demographic survey (i.e., gender, education, revenue level, marital status, kids). Prior to the FT tasks, the participants completed the DDQ in order to prevent any influence of FT on the delay discounting function. The order of the FoFTS scale and the PFT was counterbalanced across the participants. The PFT was interviewed and recorded for subsequent coding.

### 2.4 Statistical analysis

The statistical analysis was performed using the Statistical Package for Social Sciences (IBM SPSS 22. For windows). The skewness and kurtosis were generated for the normality of each variable's distribution.

Where the data met the related assumptions, the parametric tests were used (Pearson product‐moment correlation *r*), otherwise, the equivalent nonparametric tests were applied (Spearman's rank‐order correlation *rho*).

A series of simple regression analyses were conducted with the predictors as the independent variables and the DD rate as the dependent variable. In order to prevent collinearity, the highly correlated variables were not simultaneously integrated into the regression. A final multiple regression model was conducted to specify the predictive value of the predictors.

## 3 RESULTS

Due to a large amount of skewness and kurtosis for the discount rates (skewness = 3.641, kurtosis = 15.862), intrapersonal contents (skewness = 2, kurtosis = 3.649), distant intrapersonal contents (skewness = 2.284, kurtosis = 5.369), and distant errands (skewness = 2.81, kurtosis = 7.916), the statistical analyses were performed on the log‐transformed delay discounting parameter (i.e., k) and the square roots of intrapersonal contents, distant intrapersonal, and distant errands.

Prior to the analysis, the data were examined for any statistical outliers. The scores above or below three SD on variables were included in the data since no abnormalities were assigned to them.

### 3.1 Correlation analyses

**FIGURE 2:**
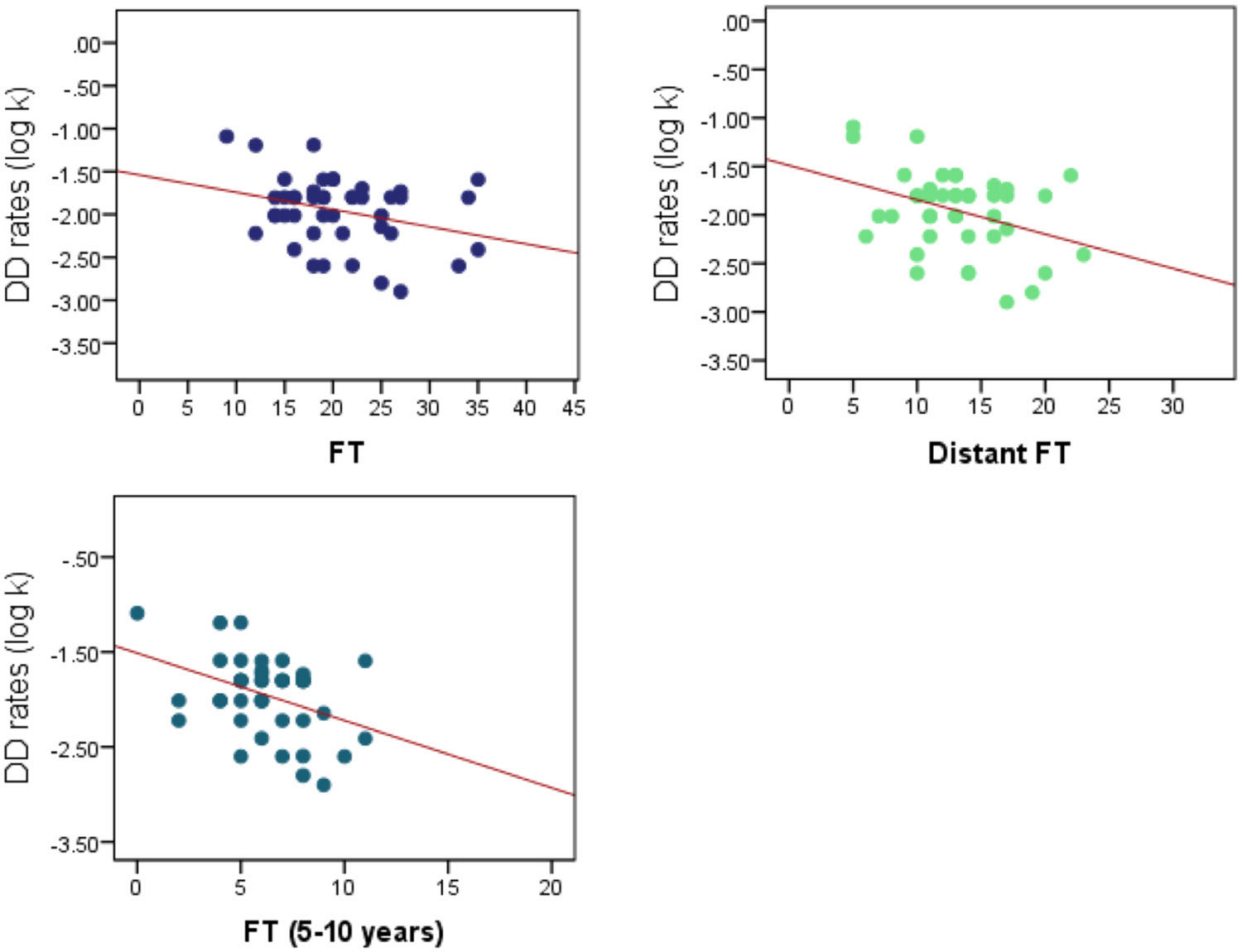
Scatterplots of the log‐transformed DD rates (Horizontal axes) and the fluency of future thinking (Vertical axes) for A: the sum of the future thoughts (FT), B: only the distant future thoughts (Distant FT), and C: future thoughts of 5–10 years later (FT (5‐10 years))

The zero‐order correlations between the independent variables and the dependent variable were performed. The results supported the hypothesis that the DD rate would be associated with and predicted by the fluency of future thoughts (Figure 2). However, the hypothesized relationship between DD and future thoughts about achievements was not proven. Table 2 presents significant correlations between the DD rate and independent variables. Since demographic factors have been studied frequently before and proven associating significantly with DD, they were also studied accordingly. Table 2 presents significant correlations between the DD rate and independent variables. By exploring partial correlations controlling for any effect of gender and education, the significant relations in Table 2 were confirmed (Table 3 ).

**TABLE 2:** Zero‐order correlations between predictors and DD

DD (Log‐transformed *k*)		Education	FT^1^	Distant FT^2^	FT (5‐10 years)	Distant financial	Planning
	*r/rho p*‐value	−0.208* 0.032	−0.307* 0.043	−0.364* 0.015	−0.389** 0.009	−0.309* 0.041	−0.223* 0.022

^1^ Total number of future thoughts.

^2^ Distant delays are considered (1 year, 5–10 years).

**TABLE 3:** Partial correlations between predictors and DD controlling for gender and education

DD (Log‐transformed *k*)		FT^1^	Distant FT^2^	FT (5‐10 years)	Distant financial	Planning
	*r p*‐value	−0.305* 0.049	−0.360* 0.019	−0.446** 0.003	−0.321* 0.038	−0.213* 0.030

^1^ Total number of future thoughts.

^2^ Distant delays are considered (1 year, 5–10 years).

**TABLE 4:** Summary and coefficients of simple regression models

Model	Significant variables	*R*	*R* ^2^	Adjusted *R* ^2^	*β*	Sig.	*F*	Sig.
FT (5–10 years)	FT (5–10 years)	0.38	0.15	0.13	−0.389	0.009	7.471	0.009
Distant FT	Distant FT	0.36	0.13	0.11	−0.364	0.015	6.398	0.015
Distant financial	Distant financial	0.309	0.095	0.074	−0.309	0.041	4.433	0.041

### 3.2 Predictive models

#### 3.2.1 Simple regression models

The total number of future thoughts, distant future thoughts, and future thoughts for the upcoming 5–10 years were highly correlated (*r* > 0.8, p < 0.001). Therefore, in order to prevent collinearity, they were not simultaneously integrated into the regression. Since the distant financial content was determined significant, it was ultimately entered in the final multiple regression (Table 4).

#### 3.2.2 Multiple regression models

As mentioned earlier, education was significantly correlated with the DD rates, and gender caused significant mean differences in the number of future thoughts (5–10 years); therefore, multiple regressions controlling for the gender and education covariates were performed. Results have been depicted in Table 5. The total number of future thoughts (5–10 years) explained the most variance of DD compared to the sum of all the future thoughts and distant future thoughts. Hence, according to the research aim, future thoughts (5–10 years) were integrated into the final multiple regression along with distant financial content.

**TABLE 5:** Summary and coefficients of multiple regression models controlling for education and gender

Model	Significant variables	*R*	*R* ^2^	Adjusted *R* ^2^	*β*	Sig.	*F*	Sig.
FT (5–10 years) + Education+ Gender	FT (5–10 years)	0.495	0.245	0.20	−0.425	0.006	4.972	0.005
	Education				−0.273	0.056		
Distant FT + Education + Gender	Distant FT	0.46	0.21	0.154	−0.302	0.032	3.613	0.021
Distant financial +Education+ Gender	Distant financial	0.46	0.21	0.156	−0.316	0.030	3.658	0.02
	Education				−0.347	0.019		

#### 3.2.3 Final multiple regression model

Since the planning was significantly correlated with the DD rates, it was entered into the final model along with other selected predictors. Final multiple regression analysis with the total number of future thoughts (5–10 years), distant financial contents, gender, education, and planning was conducted (See Tables 6 and 7 for more details). The model as a whole explained 40% of the variability in the discount rates (*R* ^2^ = 0.40). Total future thoughts (5–10 years) and distant financial contents significantly predicted the delay discounting when considering education, gender, and frequency of future thoughts for planning in the final regression model.

**TABLE 6:** Summary of final regression model

*R*	*R^2^*	Adjusted *R^2^*	*F*	Sig *F*
0.66	0.4	0.33	5.186	0.001

**TABLE 7:** Summary of final model coefficients

	*Β*	Sig.	*t*
(Constant)		0.336	0.974
FT (5–10 years)	−0.342	0.019	−2.451
Distant financial	−0.270	0.045	−2.068
Education	−0.262	0.05	−2.025
Planning	−0.296	.026	−2.311
Gender	−0.170	.227	−1.227

## 4 DISCUSSION

The present study investigated the relationship between FT and delay discounting considering fluency, contents, and functions of future thoughts. Delay discounting describes intertemporal decision‐making, as a predictor of impulsive behavior, less patience, and lack of future‐oriented behaviors (Green and Myerson, 2004; Mazur, 1987). The results implied a negative relationship between the frequency of future thoughts one can generate and the delay discounting. In other words, the individuals who generated more future thoughts discounted less, suggesting them being future‐oriented. The results were more promising for thoughts of the distant future (i.e., 1 year and 5–10 years later), as better FT for an upcoming year or 5–10 years later was associated with less shortsighted intertemporal decisions.

One possible explanation for the observed relationship is that individuals who can generate more future thoughts would be able to rapidly produce future mental representations when facing an intertemporal choice. Indeed, they can specify more value and time to the future and represent the future, as it is near and accessible, therefore, discounting the delay to a lesser extent. These findings were consistent with previous studies showing that individuals with more vivid episodic future thoughts project less discounting behaviors (Bromberg et al., 2015). In addition, the present study extends the understanding of the relationship between intertemporal decision‐making and FT by considering the direct and fast mode of generating future thoughts regardless of specificity and vividness in episodic future representations. Since the individuals in daily decision‐making are not usually forced to make novel episodic future thoughts as in the laboratory, it is valuable to consider the direct mode of FT in order to generalize its application to delay discounting in real ecological settings. The contribution of episodic FT to delay discounting had been widely studied (Dassen et al., 2016a; Nan & Qin, 2019; O'Donnell et al., 2017, 2017, 2019; O'Neill et al., 2016; Stein et al., 2017, 2016, 2021; Rosch et al.,^2022^) indicating less discounting behavior when a cue is presented to the participants (i.e., their own episodic future thoughts) while performing the delay discounting task. Direct generation of future thoughts may serve the stream of studies by producing a more sustained effect in daily decision‐making.

When considering the frequency of future thoughts per category, the financial contents of future thoughts were associated with patience in favor of delayed rewards. This finding may be due to the modality of the delay discounting task accounting for monetary rewards. Prior studies (O'Donnell et al., 2017) showed a reduction in delay discounting in participants exposed to some FT cues which were amplified when the cues matched their financial goals. The present study indicated that the total amounts of future thoughts, as well as the amount of financial future thoughts, were associated with more future‐oriented behaviors. By controlling the regression models for gender and education, models that are more significant were achieved.

In line with previous research, higher education is associated with less impulsive behavior (Jaroni et al., 2004; Reimers et al., 2009). Jaroni et al. (2004) suggested that larger rewards in education are mainly delayed unless the participant fails in gaining any educational achievement.

Taking a functional approach, more frequent FT for planning functions was associated with less tendency to immediate rewards. The results are interesting since planning requires following a procedure in mind in order to achieve a particular outcome (Morris & Ward, 2004). Individuals, who frequently imagine the necessary steps to accomplish a plan, would more often consider a delayed reward.

Although we anticipated frequent thoughts about future achievements being associated with delay discounting, the results did not provide strong support for such relationship. This may highlight the importance of the functions while thinking about the future regardless of the contents of thoughts. For instance, individuals may generate many thoughts about their leisure activities, but as long as they are thinking about planning, they contribute to diminishing discounting behaviors.

Categorizing future thoughts in the present study was conducted based on prior studies. However, a more comprehensive study yet deviated from the mainstream research should be performed with the participants generating future thoughts separately for each category. It is also suggested that valence, arousal, and personal relevance of the future contents should be considered for each future thought generated. Given the previous findings on thematic contents of future thoughts on some behaviors and disorders (Bouwman, 2016; Godley et al., 2001; O'Connor et al., 2015), it is crucial to investigate a unitary system for grouping future contents considering essential aspects of human life.

## 5 CONCLUSION

This study examines whether the delay discounting varies as a function of FT, contents of future thoughts, and functions inspiring FT. Findings indicate a negative relationship between the delay discounting and fluency of future thoughts, representing that frequently generating future thoughts is associated with less discounting behavior. This relationship is enhanced for longer delays (e.g., 5–10 years). Likewise, the financial category among thematic contents of future thoughts was associated with lower delay discounting. The final proposed model proves the predictive value of fluency and financial contents of future thoughts in delay discounting when controlling for education, gender, and accounting for planning.

## CONFLICT OF INTEREST

The authors declare no conflicts of interest to disclose.

### PEER REVIEW

The peer review history for this article is available at: https://publons.com/publon/10.1002/brb3.2764.

## Data Availability

The data that support the findings of this study are available upon reasonable request. The data are not publicly available due to ethical restrictions regarding the privacy of research participants.
